# Accuracy of dose planning for prostate radiotherapy in the presence of metallic implants evaluated by electron spin resonance dosimetry

**DOI:** 10.1590/1414-431X20154367

**Published:** 2015-05-26

**Authors:** G.G. Alves, A. Kinoshita, H.F. de Oliveira, F.S. Guimarães, L.L. Amaral, O. Baffa

**Affiliations:** 1 Departamento de Física, Faculdade de Filosofia, Ciências e Letras de Ribeirão Preto, Universidade de São Paulo, Ribeirão Preto, SP, Brasil; 2 Universidade Sagrado Coração, Bauru, SP, Brasil; 3 Serviço de Radioterapia, Hospital das Clínicas, Faculdade de Medicina de Ribeirão Preto, Universidade de São Paulo, Ribeirão Preto, SP, Brasil

**Keywords:** Alanine electron spin resonance dosimetry, Prostate, Electron paramagnetic resonance, Treatment-planning system, Inhomogeneity correction

## Abstract

Radiotherapy is one of the main approaches to cure prostate cancer, and its success depends on the accuracy of dose planning. A complicating factor is the presence of a metallic prosthesis in the femur and pelvis, which is becoming more common in elderly populations. The goal of this work was to perform dose measurements to check the accuracy of radiotherapy treatment planning under these complicated conditions. To accomplish this, a scale phantom of an adult pelvic region was used with alanine dosimeters inserted in the prostate region. This phantom was irradiated according to the planned treatment under the following three conditions: with two metallic prostheses in the region of the femur head, with only one prosthesis, and without any prostheses. The combined relative standard uncertainty of dose measurement by electron spin resonance (ESR)/alanine was 5.05%, whereas the combined relative standard uncertainty of the applied dose was 3.35%, resulting in a combined relative standard uncertainty of the whole process of 6.06%. The ESR dosimetry indicated that there was no difference (P>0.05, ANOVA) in dosage between the planned dose and treatments. The results are in the range of the planned dose, within the combined relative uncertainty, demonstrating that the treatment-planning system compensates for the effects caused by the presence of femur and hip metal prostheses.

## Introduction

Prostate cancer is the most common type of cancer in men, and the median age of diagnosis is 72 years (1). An estimated 233,000 new cases will be diagnosed in 2014, accounting for 27% of new cancer cases in men in 2014 (2). Estimates of life expectancy have emerged as a key determinant of primary treatment, particularly when considering active surveillance or observation. Radical prostatectomy is appropriate for any patient whose tumor is clinically confined to the prostate. However, because of potential perioperative morbidity, radical prostatectomy should be reserved for patients whose life expectancy is 10 years or more. Over the past several decades, radiation therapy techniques have evolved to allow higher doses of radiation to be administered safely. Three-dimensional conformal radiation therapy (3D-CRT) uses computer software to integrate computerized tomography images of the patient's internal anatomy in the treatment position, which allows higher cumulative doses to be delivered with lower risk of late effects (3-6). The second-generation 3D technique, intensity-modulated radiation therapy (IMRT), is used increasingly in practice (7). When compared with 3D-CRT, IMRT significantly reduces the risk of gastrointestinal toxicities and rates of salvage therapy without increasing side effects, albeit with an increase in treatment cost (8-10).

As the population ages and the use of hip prostheses becomes more common, the problem of treating patients with prostheses will also increase (11). Despite the increase in the number of patients using metallic prostheses, several studies show problems with the current planning system related to dose calculation because of the presence of materials with electronic densities differing from water.

Pasciuti et al. (12) showed problems with the algorithms used in IMRT planning in the presence of heterogeneities. The results showed errors in dose calculation involving lungs when the pencil beam algorithm was performed, while collapsed cone convolution superposition and the anisotropic analytical algorithm showed a higher degree of accuracy. The Intensity Modulated Radiation Therapy Collaborative Working Group (13) suggested using a correction for heterogeneity mainly in lung treatment and suggested exercising caution when dealing with cases involving materials that can cause artifacts, such as those with metal prostheses. Thus, these studies showed that planning systems should be tested and validated for different settings, especially for settings that involve a material with a high atomic number.

For patients with metallic hip prostheses, treatment planning has to be performed with consideration of the prosthesis material as well as its position and form if photon beams are to be administered through the prosthesis. However, knowledge about the prosthesis material may be missing, or the actual geometry may deviate from the assumed one, resulting in serious under- or overdosage of the tumor, or an increase in dose to at-risk organs, thus compromising tumor control and producing severe collateral problems for the patient.

Because there are few studies to validate treatment-planning systems, this study was conducted to investigate the accuracy of dose planning in prostate cancer for cases involving patients with metallic prostheses. A home-made phantom of a pelvic region with the dimensions of an adult human and electron spin resonance (ESR) dosimetry with alanine as a dosimeter were used. Alanine has desirable features such as: tissue equivalence, a linear relationship in the dose range of interest, no energy dependence for photons above 100 keV, dose rate independence, small temperature dependence during irradiation, and other characteristics that allow for the precise elucidation of the issues involved in dose planning for this study (14-16).

## Material and Methods

### Dosimeters

The dosimeters used in this study were made in our laboratory and were composed of 95% DL-alanine (Sigma-Aldrich, USA) and 5% polyvinyl alcohol (Sigma-Aldrich), with a total mass of 50 mg, compressed to form pellets 3 mm in diameter and 4 mm in length, in accordance with the procedures described by Chen et al. (17). A set of dosimeters was produced in sufficient quantity for the entire experiment.

To test the response homogeneity of the batch of dosimeters produced, 25 dosimeters were arranged in five rows and five columns in a 30 x30 cm phantom made of solid water, with 1 cm internal spacing, and with the appropriate built-up and backscatter layers. The phantom was irradiated with a dose of 2 Gy of a 6-MV X-ray beam (Oncor Impression linear accelerator, Siemens, Germany) with 1.5 cm solid water to establish electronic equilibrium. After the homogeneity response test was passed by the batch of dosimeters, a subset of 50 dosimeters, five for each dose point, was irradiated with doses up to 10 Gy to build a calibration curve. The given dose was calibrated daily using an ionization chamber according to the International Atomic Energy Agency (IAEA) protocol, with a deviation of less than 1%.

The ESR spectrum of irradiated dosimeters was recorded with a JEOL FA200 X-band spectrometer (Japan) at room temperature, at least 72 h postirradiation with the following parameters: 2 mW microwave power, 348 mT central field and sweep width of 10 mT, a modulation amplitude of 0.6 mT, a time constant of 0.3 s, and a scan time of 1 min. A total of 10 scans were accumulated to improve the signal/noise (S/N) ratio. The peak-to-peak amplitude of the central line of the spectrum was used as the reading of the dosimeter to correlate the dose with the number of spins created by irradiation.

### Phantom

**Figure 1:**
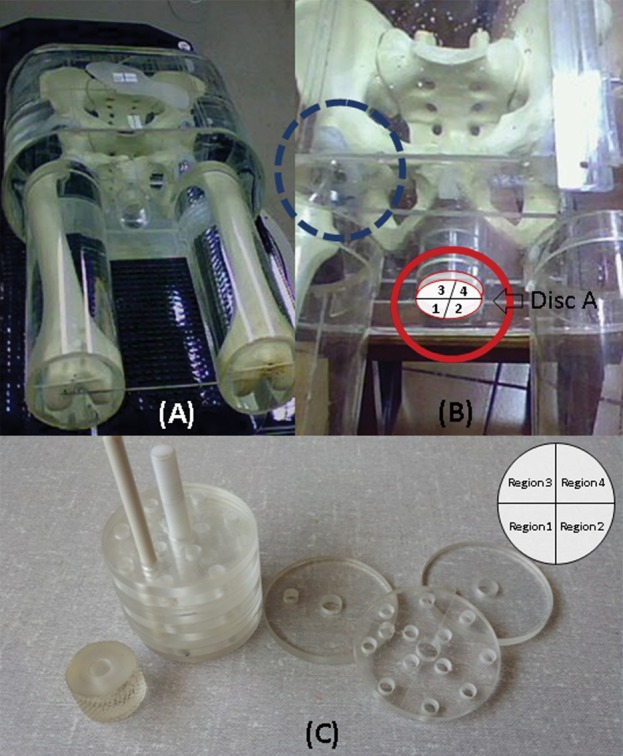
*A*, General view of the phantom. *B*, Detail of volume where the dosimeters are inserted (red circle) and the prosthesis (blue circle). *C*, Dosimeter holder composed of acrylic discs with holes to contain the alanine dosimeters and how the quadrants of the disc were labeled.

A phantom of the pelvic region with the dimensions of an adult human was made using an acrylic vessel filled with water to simulate soft tissue. Human bones and/or prostheses were used in the femur and hip positions (Figure 1A). The prostheses were composed of a polyethylene acetabular component and a stainless steel femoral stem (Baumer, Brazil). The bone cement used was composed of methyl/polymethyl methacrylate and a radiopaque agent, the same cement employed for hip prosthesis implants by the surgical team of the Departamento de Ortopedia do Hospital das Clínicas, Faculdade de Medicina de Ribeirão Preto, Universidade de São Paulo.

In the prostate region, a cylindrical opening, 4.4 cm in diameter and 5.0 cm in length, was made to insert the alanine dosimeters (Figure 1B). Acrylic discs 4.4 cm in diameter were made to hold 12 dosimeters. Four discs (A, B, C, and D) were stacked, interspersed with a 1-cm layer, forming a cylinder occupying the whole region of the prostate (Figure 1C). Each disc was divided into four regions (1 to 4) for better control of the spatial distribution of the dose (Figure 1C). This cylinder was inserted into the prostate region, with disc D in the innermost region of the phantom, and with regions 1 and 2 of the discs oriented toward the posterior part of the phantom.

### Radiotherapy planning of prostate cancer and ESR dosimetry

Simulations of irradiation were carried out using the tomographic images of the phantoms in the treatment-planning system (TPS) XiO (version 3.62, Elekta AB, Sweden), with four fields having gantry angles of 0, 90, 180, and 270 degrees, respectively. All the dosimeters were positioned between isodose curves of 99 and 101%. The calculation algorithm used was superpositioned with a 2-mm calculation grid, using all simulations of heterogeneity correction and incorporating the electronic density of all materials involved. The dose was planned using the CT imaging obtained from the phantom with and without the prosthesis as follows: phantom empty (acrylic and water only), phantom without prosthesis (with bones in the femur position), phantom with one prosthesis (unilateral), and phantom with two prostheses (bilateral).

Irradiation was performed with an Oncor Impression linear accelerator (Siemens) using an X-ray beam of 6 MV with a dose of 2 Gy, given in four fields of irradiation with dimensions 10 x10 cm^2^, in accordance with the planning system. The phantom was positioned through the displacement as indicated by the TPS, between the reference marks made during CT with radiopaque markers and the isocenter coordinates of the simulation.

## Results and Discussion

### Homogeneity of dosimeters and calibration curve

**Figure 2:**
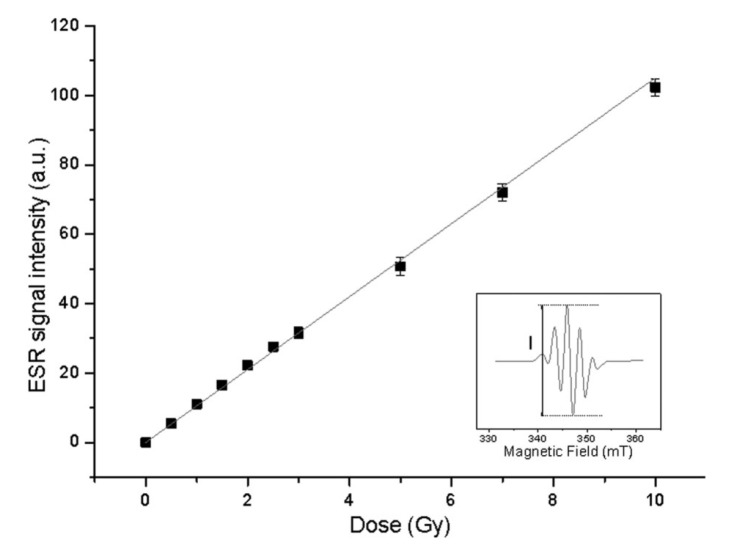
Calibration curve for the electron spin resonance (ESR)/DL-alanine dosimeter. The inset shows the typical spectrum and the amplitude of the central line h used for the calibration. (Pearson's r = 0.9994; Instrumental weighting).

Twenty-five dosimeters were irradiated with 2 Gy, and the measured amplitude of the ESR signal had a 5% deviation, with a good agreement within the given relative combined standard uncertainty and within the accepted tolerance for planning doses for the whole procedure of dosimeter production, positioning during irradiation, and spectrum recording of radiotherapy (18). Although the precision and homogeneity of dosimetry can be improved with higher doses, in this study a dose of 2 Gy was used to simulate the typical dose given at a session of radiotherapy. The calibration curve (Figure 2) demonstrates a linear relationship between the amplitude I and dose D given by equation 1, where I is the amplitude in arbitrary units given by the ESR spectrometer (usually a voltage converted into units of the digitizer), normalized by the mass of each dosimeter.

(Eq.\ 1) I=(10.5±0.1).D

### Radiotherapy planning of prostate cancer

**Figure 3:**
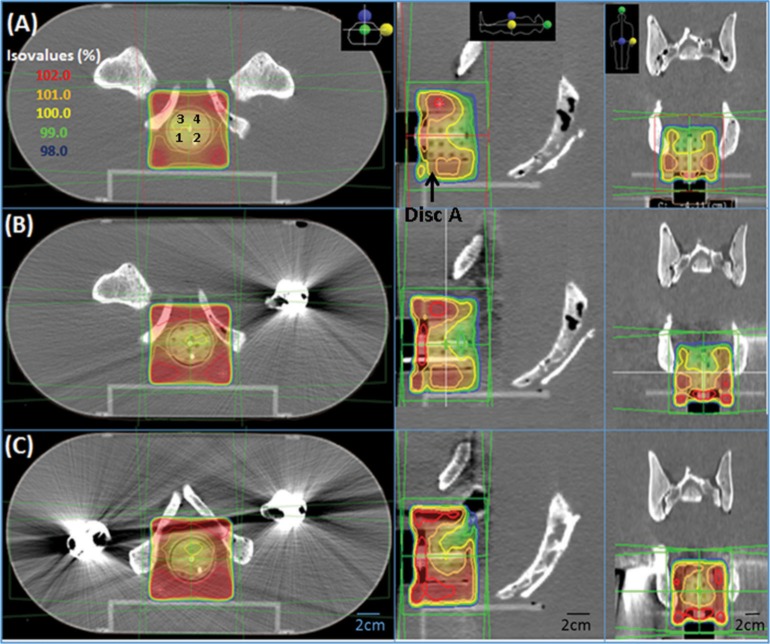
Treatment plan images showing the isodose curves in the prostate region in the axial section (left) and sagittal (center) and coronal regions (right), planned according to the following conditions: without prosthesis (*A*), unilateral prosthesis (*B*) and bilateral prostheses (*C*). The icons at the top indicate the orientation of the images in relation to the human body.

Figure 3 shows the isodose curves in the prostate region in the axial section (left) and sagittal (center) and coronal regions (right), planned according to the following conditions: without prosthesis (A), unilateral prosthesis (B), and bilateral prostheses (C). These results show that the dose-planning system ensured that all dosimeters were positioned between isodose curves of 99 and 101%.

In Figure 3C, it can be observed that the posterior region of the phantom received a higher dose; this result is expected because the anterior region is composed of tissues of higher density, because the metallic prostheses are in the lateral projections of irradiation fields. This fact was also observed via the sagittal view, where the dose is more intense at the caudal than at the cranial region.

### ESR dosimetry

**Figure 4:**
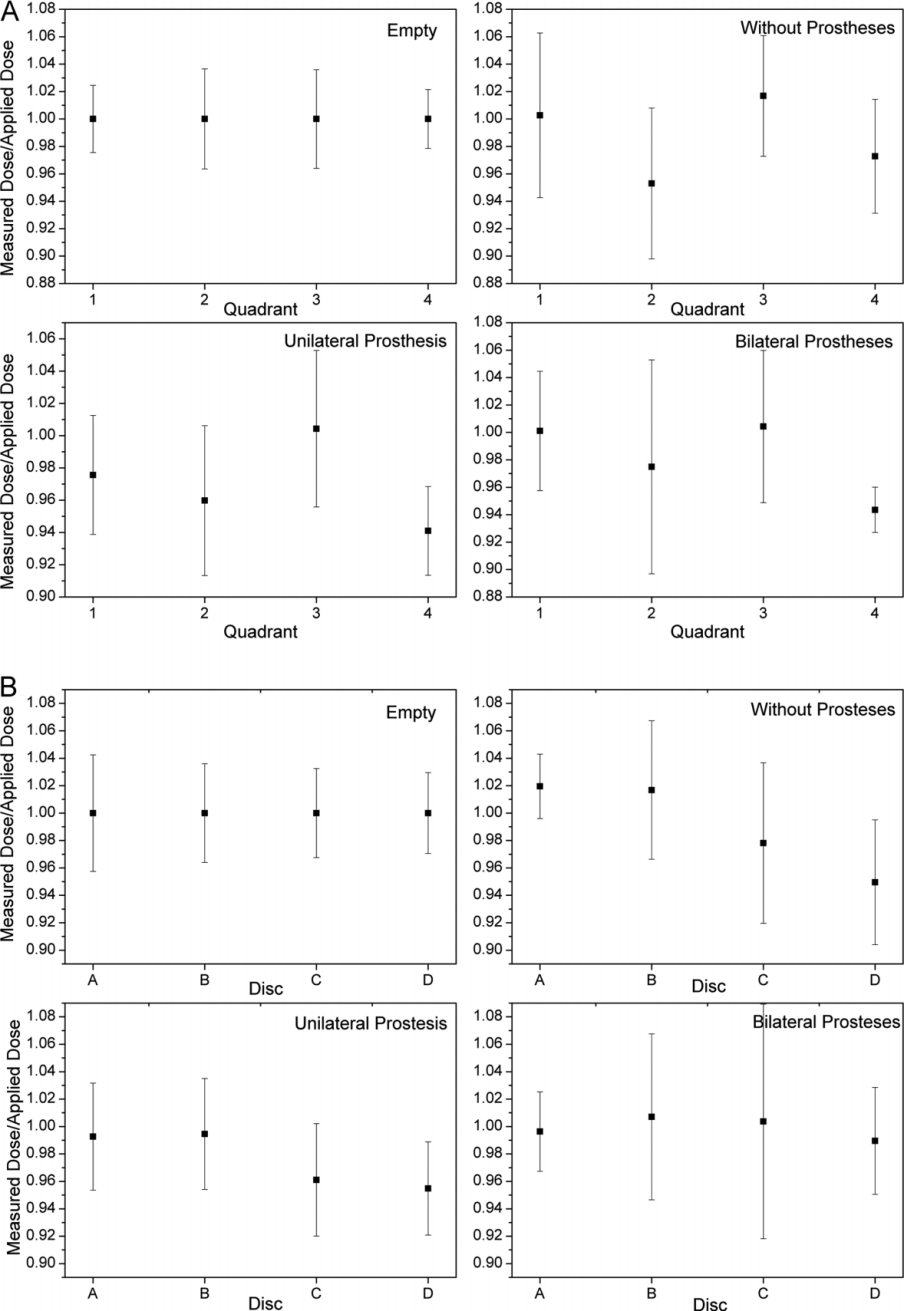
Results of the ratio measured dose/applied dose obtained for the 4 cases studied and different positions in the phantom according to the quadrant (*A*) and to the disc (*B*). There were no differences in the results (P>0.05, ANOVA).

Figure 4 shows the results of dosimetry obtained after irradiation in the phantom under the described situations. The results are reported as the ratio of measured to applied doses. Although the results did not present statistically significant differences, the average doses of the quadrant 4 region were the lowest found. This is in agreement with isodose simulation, which demonstrates lower doses in this region (anterior) of the phantom. In addition, considering the standard deviation, the region of discs B and C for the case of bilateral prostheses may exceed the maximum limit of 5%. The false CT numbers noted in Figure 3 were not corrected in this case to effectively evaluate the performance of the software without external intervention. In these cases, one usually has to give the correct Hounsfield units (HU) value to the regions surrounding the metallic prostheses and soft tissue of interest. The results showed that this was not a critical issue in this case. If the planning target volume (PTV) were closer to the prostheses, the correction would be necessary.

The *in vivo* study conducted by Wagner et al. (19) also used alanine dosimeters to check the dosimetry for prostate IMRT treatment. In contrast to the results of this study, they found differences a little higher in the TPS and ESR dosimetry for patients with metallic prostheses. The dose at the anterior rectal wall was overestimated by the TPS by approximately 11%, and the dose at the posterior rectal wall was underestimated by approximately 7%. These findings show the importance of having a deep knowledge of the performance of the TPS in different situations, and phantom studies may serve as a guide.

### Uncertainty estimation

As explained by Wagner et al. (20), because the ratio of measured to applied dose (by means of the TPS including the dose delivery on the accelerator) was considered as the end result, the following sources contributing to the overall uncertainty of the result were identified: measurement method of alanine/ESR, daily fluctuation of the accelerator output in terms of dose/monitor unit (MU) under reference conditions, dose calculation of the TPS, and positioning of the alanine dosimeters.

Wagner et al. (20) also considered the irradiation temperature and fading corrections due to high temperature of irradiation as a feature of *in vivo* studies. In this study, it was not necessary because studies were performed on a phantom.

The components of uncertainty for the applied dose are given in Table 1, taking into account the daily fluctuation of machine output in terms of dose/MU, the dose calculation of the TPS, phantom positioning, and alanine positioning. All uncertainties are given as k=1 (1σ) standard uncertainties.

**Table 1:**
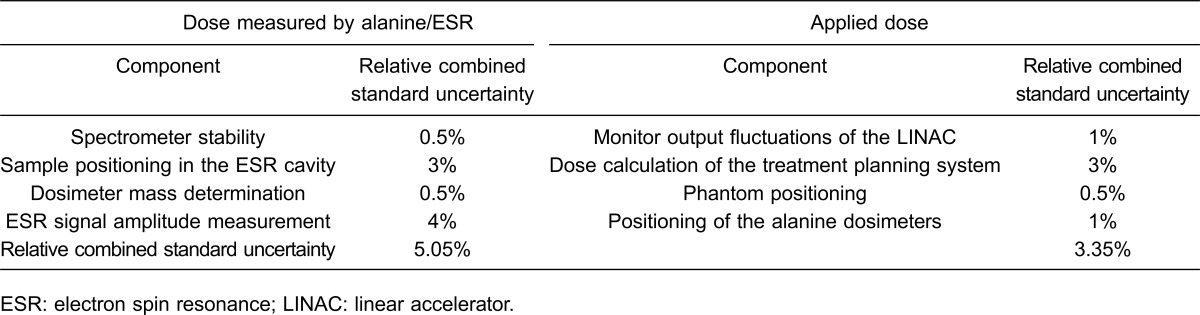
Uncertainty estimation.

The measured dose by the ESR/alanine system presented an overall variation of 5.05% that integrates the fluctuations due to sample positioning in the cavity (3%), error in the sample mass determination (0.5%), peak-to-peak measurement (4%), and the stability of the ESR spectrometer.

In conclusion, ESR dosimetry with alanine shows that the planning system with the correction of inhomogeneity of the materials adequately resolves the attenuation effect caused by metal prostheses and gives the expected results in an experiment using a phantom pelvic region.
